# Learning curve for transcatheter aortic valve replacement for native aortic regurgitation: Safety and technical performance study

**DOI:** 10.1002/clc.23332

**Published:** 2020-01-11

**Authors:** Lulu Liu, Jian Zhang, Ying Peng, Jun Shi, Chaoyi Qin, Hong Qian, Zhenghua Xiao, Yingqiang Guo

**Affiliations:** ^1^ Department of Cardiovascular Surgery, West China Hospital Sichuan University Chengdu China; ^2^ Department of Cardiology, West China Hospital Sichuan University Chengdu China

**Keywords:** learning curve, native aortic regurgitation, transcatheter aortic valve replacement

## Abstract

**Background:**

Transcatheter aortic valve replacement (TAVR) is a fundamentally new procedure for the treatment of native aortic regurgitation (AR). The number of cases needed to gain proficiency with the procedure is unknown.

**Hypothesis:**

This study aimed to evaluate the learning curve for TAVR for native AR.

**Methods:**

This study retrospectively reviewed a prospective database from 134 consecutive native AR patients who underwent the J‐valve TAVR system, which performed by a single team interventional cardiologist. The cumulative sum (CUSUM) method was used to analyze the learning curve. Patients were divided into two groups in chronological order, defined by the surgeon's early (group 1: the first 52 cases) and skilled (group 2: the next 82 cases) experience. Demographic data, intraoperative characteristics, and short‐term surgical outcomes were compared between the two groups.

**Results:**

CUSUM plots revealed decreasing procedure time and fluoroscopy time after patients 52 and 43, respectively. The patient date consistently demonstrated that high‐risk scores and major perioperative parameters were comparable between the two groups. The use of contrast dye (group 1, 94.22 ± 30.07 mL; group 2, 70.43 ± 15.02 mL, *P*<.05), total procedure time (group 1, 84.96 ± 17.76 minutes; group 2, 59.95 ± 12.83 minutes, *P*<.05), and fluoroscopy time (group 1, 11.52 ± 3.81 minutes; group 2, 6.47 ± 1.53 minutes, *P*<.05) were significantly reduced in group 2. The overall device success rate in group 1 was 96.2% vs 96.3% in group 2 and remained high (*P* = 1.0). The overall 30‐day mortality was 3.8% in group 2 (group 1, 0 to group 2, 3.8%; *P* = .16). The complications rate, such as pulmonary hypertension, chronic kidney disease, and coronary artery disease were higher in group 2.

**Conclusions:**

For a surgeon without previous TAVR experience, 52 cases of performance is the minimal requirement to gain the proficiency of TAVR for native AR. The skilled surgeons have been observed with reduced procedural time, fluoroscopy times, radiation exposure dose, and contrast volume usage. However, the overall prognosis was not significantly different between the two groups.

## 1 INTRODUCTION

Transcatheter aortic valve replacement has gained increasing acceptance as a treatment option for patients with severe symptomatic aortic stenosis (AS) who are considered low to high risk for surgical aortic valve replacement (SAVR).^1^, ^2^, ^3^ Some learning curve studies have shown that it takes approximately 30 cases for a team to become proficient in performing the transcatheter aortic valve replacement (TAVR) procedure on severe calcified AS patients.^4^, ^5^ According to the Euro Heart Survey, one in five patients with native valve disease suffers from pure aortic regurgitation (AR).^6^ However, Pan et al reported that AR is more prevalent in the elderly Chinese population.^7^ Thus, the J‐valve, JenaValve, and Medtronic Engager devices have been specifically designed for the treatment of AS and aortic insufficiency.

Transcatheter aortic valve replacement is a technically demanding and time‐consuming surgical procedure; thus, evaluation of the learning curve for TAVR in patients with AR is necessary to guide the training of novice surgeons working to adopt this new technique. Limited data are available regarding the learning curve of TAVR in the treatment of patients with native AR. The aim of this study was to evaluate the learning curve for TAVR in the treatment of patients with native AR, which may improve the possible introduction of this technology for use with AR individuals in the future.

## 2 MATERIALS AND METHODS

### 2.1 Study design

The procedure was performed with only one surgeon as the operator; the surgeon had no previous experience in the TAVR procedure. From April 2014 to April 2019, 134 high‐risk patients with native AR underwent TAVR with the J‐valve prosthesis by a single surgical team in the West China Hospital Department of Cardiovascular Surgery, Sichuan University. All patients were preoperatively diagnosed with native AR by echocardiographic images and underwent a systematic evaluation, including coronary angiography and doppler echocardiography. Computed tomography was performed to determine the valve size and anatomy of the apical approach. The study protocol was approved by the West China Hospital Ethics Committees and Institutional Review Board, Sichuan, China. Written informed consent was obtained from each of the enrolled patients.

### 2.2 Patients

The indications in our study were identical to those for SAVR, including some challenging cases. Inclusion criteria included echocardiography determined moderate‐severe or severe pure AR; age ≥60 with heart function grade of NYHA II‐IV; electrocardiography results indicating more than moderate regurgitation, irreversible complications; or other influential postoperative factors or high risk for surgery (STS≥8); aortic annulus >19 and <29 mm, standardized using cardiac CT measurements; and ascending aortic diameter <50 mm. The exclusion criteria were as follows: moderate or mild AR; previous history of endocarditis or active endocarditis; diameter of annulus >29 mm or <18 mm; severe hemorrhagic tendency, contraindications for anticoagulation therapy or anticoagulation taboo; onset of Transient Ischemic Attacks or severe stroke or severe dementia within 6 months; myocardial infarction within a month; any cardiac mass discovered during echocardiography, left ventricular, or atrial thrombosis; unstable hemodynamics, or need for high doses of cardiostimulatory drugs or mechanical cardiac assistance; hypertrophic cardiomyopathy; severe disease with life expectancy ≤1 year; potential aortic stenosis defined as peak aortic jet velocity >2.5 m/s identified on continuous‐wave color Doppler ultrasound.

### 2.3 Study device and procedure

The high incidence of valve migration and paravalvular leakage (PVL) prevents the widespread use of TAVR for the treatment of patients with AR. The J‐Valve system (JieCheng Medical Technology Co., Ltd., Suzhou, China) is a self‐expanding transcatheter heart valve with a unique two‐piece structure design that consists of three U‐shaped graspers encircling the valve stents. The J‐valve was certified for AR and AS by the China Food and Drug Administration in 2017. The learning curve was established for TAVR in patients with pure AR using the J‐valve only in our center, for surgeons without any prior experience with the TAVR procedure.

**Figure 1:**
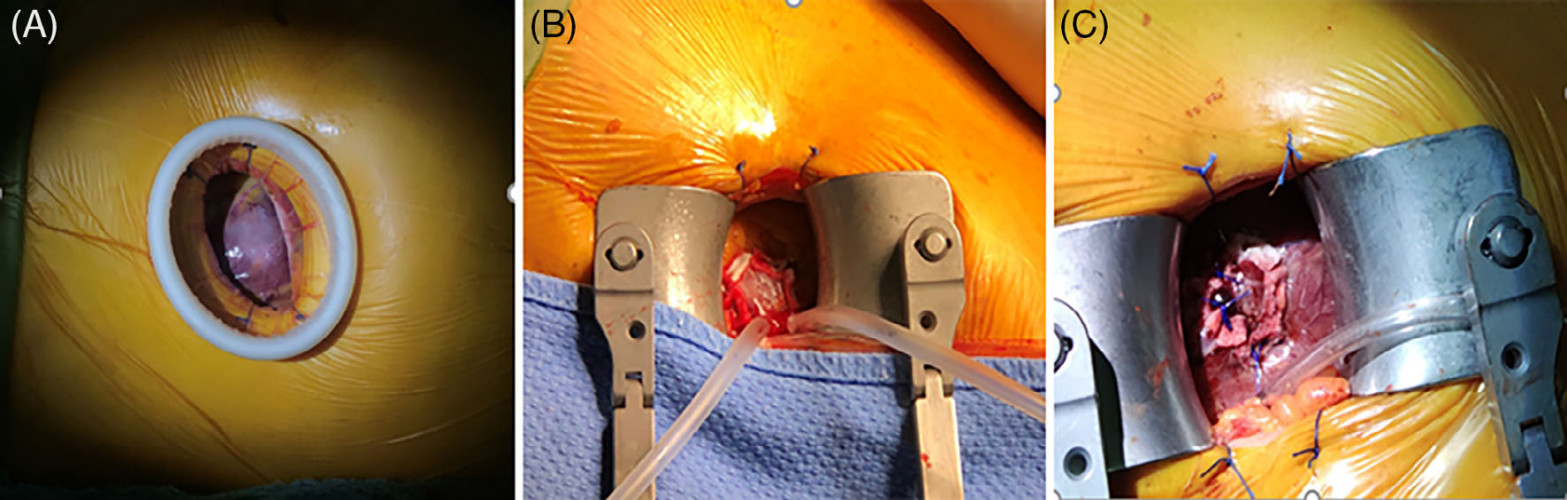
Apical approach; left. A, 3 to 5 cm intercostal incision exposes the left ventricular apex. B, Teflon‐reinforced mattress double circular sutures were placed on the left ventricle apex. C, the left ventricle apex is sutured after valve implantation

**Figure 2:**
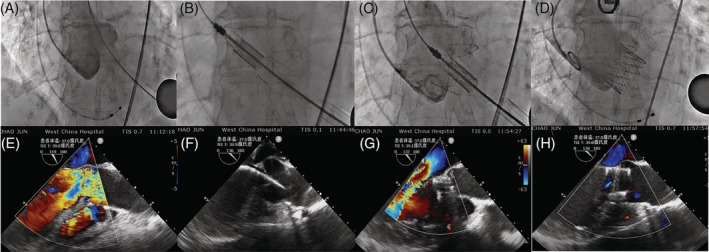
Intraoperative angiogram and TEE images of TAVR procedure by J‐Valve system with pure aortic regurgitation. A, Aortic root angiogram to set up optimal C‐arm angulation. B, Prosthesis was inserted above aortic annulus to the ascending aorta and locators were deployed. C, Prosthesis descended to the annulus level and aortic root angiogram to verify the position of prosthesis and locators. D, Successful prosthesis release and postoperative aortic root angiogram again. TEE during the procedure: E, TEE of native aortic valve severe regurgitation before THV implanted. F, TEE of prosthesis was inserted above aortic annulus; G, TEE prosthesis descended to the annulus level; H, TEE of aortic stent valve well positioned after THV implanted. TEE, transesophageal echocardiography; THV, transcatheter heart valve

All procedures were performed in a fully equipped “hybrid OR” by an interdisciplinary team. A full cardiopulmonary bypass circuit was on standby in all cases. An incision of 3 to 5 cm in the corresponding costal space at the left apex of the heart was made (Figure 1A). Two 3‐0 polypropylene (Ethicon, Somerville, New Jersey) Teflon‐reinforced double loop mattress sutures were placed on the left ventricular apex (Figure 1B). The delivery system was bluntly inserted into the left ventricle through the apex and advanced into a supra‐annular position over an Amplatz Super stiff guidewire (Boston Scientific; Figure 2A,B). During the first stage, the clasper was positioned into the aortic sinuses, and an angiogram was performed to confirm the correct position (Figure 2C). In the second stage, the valve was gently retrieved back into the annular plan with the guidance of the claspers and deployed without rapid ventricular pacing. After retrieval of the delivery system, a repeat aortic root angiography revealed PVL and patent coronaries as well as an optimal valve stent position (Figure 2D). TEE was also used to confirm the valve function during the procedure (Figure 2E‐H). Details of the technique details have been previously described.^8^ The analysis of the learning curve refers to transapical TAVR with AR experience.

### 2.4 Study endpoints

The primary endpoints of this study were all‐cause mortality at 30 days post procedure. Secondary efficacy endpoints included valve hemodynamics at discharge. The safety outcomes at 30 days included VARC‐2‐defined endpoints of stroke, life‐threatening or disabling bleeding, stage 2 or 3 acute kidney injury, coronary artery obstruction, major vascular complications, and valve‐related dysfunction requiring a repeated procedure, as well as permanent pacemaker implantation rates.^9^

### 2.5 Cumulative sum (CUSUM) analysis

Process measures, such as procedure times and radiation exposure were chosen as markers for increased procedural proficiency. Based on the cross point of the CUSUM plots, the first 52 patients who underwent TAVR were assigned to group 1 (early‐experience group), and the other 82 patients were assigned to group 2 (late‐experience group). Prospectively collected data included procedure time, fluoroscopy time, valve hemodynamics, postoperative complications, and postoperative hospital length of stay.

Total surgical time was defined as the time from the start of the chest incision to the completion of closure of the wound (Figure 1C).

## 3 STATISTICAL ANALYSIS

Continuous variables following a normal distribution are presented as the mean ± SD. Continuous data were compared by unpaired Student's *t*‐test. *P* values <.05 were considered to indicate a significant difference. Categorical variables are presented as counts and percentages. A chi‐squared test or Fisher's exact test was used to compare the distribution of categorical variables between groups. All data were processed using the Statistical Package for Social Sciences, version 23 (SPSS, Chicago, Illinois).

To detect early trends in the data, the cumulative sum method is the most powerful and valuable technique.^10^ We use the CUSUM plots to analyze the learning curve for TAVR in patients with AR. To evaluate learning curves, CUSUM analysis of procedure times was used in this study. We selected the mean of the data points as a reference value. Then, this reference value was subtracted from each data point in succession, and any remainder was added to the previous sum. The resulting curve ran parallel to the x‐axis when the procedure times were as expected, rose when the procedure times were higher than expected, and fell when favorably low procedure times were observed. A learning curve was considered complete when a point for decreasing surgical time was observed from the CUSUM plot.

## 4 RESULTS

A total of 134 native AR patients underwent the TAVR procedure in this study. All patients had grades above a moderate level with symptoms of left ventricular dysfunction. One underwent SAVR due to moderate PVL and congestive heart failure 1 week after the operation, four converted to SAVR due to valve migration into the aortic arch and left ventricle and coronary obstruction. Baseline characteristics are shown in Table 1. Patients in the two groups were comparable with regard to age, sex, preoperative comorbidities, and histology. Patients demonstrated a consistently high‐risk profile throughout the study. The distribution of cardiac risk factors showed no significant difference between the two groups (STS 9.17% ± 4.54% vs 10.23% ± 5.81%, *P* = .24); logistic EuroSCORE II (10.62% ± 5.28% vs 12.16% ± 7.52%, *P* = .17). The left ventricular ejection fraction (LVEF), pulmonary hypertension, coronary artery disease, and renal insufficiency were higher in group 2 than in group 1 (*P* < .05).

**Table 1:** Baseline characteristics of patients

	Group 1 (n = 52)	Group 2 (n = 82)	*P*
	No. of patients/mean	No. of patients/mean	
Age (years)	73.2 ± 4.4	73.1 ± 7.3	.94
Gender			.93
Male	39 (75.0%)	61 (74.4%)	
Comorbidities			
Chronic obstructive pulmonary disease	27 (51.9%)	46 (56.1%)	.63
Hypertension	30 (57.7%)	58 (70.7%)	.12
Diabetes mellitus	9 (17.3%)	15 (18.3%)	.88
Cerebrovascular disease	19 (36.5%)	25 (30.5%)	.34
Pulmonary hypertension	12 (23.1%)	33 (40.2%)	.04
Hepatic cirrhosis	1 (1.9%)	0 (0)	.39
Atrial fibrillation/flutter	11 (21.2%)	21 (25.6%)	.56
Chronic kidney disease	6 (11.5%)	24 (29.3%)	.02
Coronary artery disease	12 (17.5%)	33 (40.2%)	.04
Prior PCI	3 (5.8%)	1 (1.2%)	.13
Prior CABG	0	1 (1.2%)	.42
Prior cardiac surgery	0	5 (6.2%)	.16
Previous permanent pacemaker implantation	2 (3.8%)	3 (3.7%)	1.0
Echocardiographic data			
AR grade			.17
Moderate‐Severe	13 (25.0%)	11 (14.1%)	
Severe	39 (75.0%)	67 (85.9%)	
LVEF, %	55.52 ± 11.24	50.12 ± 13.09	.02
LVEF<50%, n	18 (34.6%)	36 (43.9%)	.28
Peak aortic valve velocity, m/s	2.08 ± 0.39	2.05 ± 0.4	.66
LVEDD, mm	64.62 ± 8.55	65.52 ± 9.02	.56
LVESD, mm	44.87 ± 9.52	47.89 ± 11.40	.11
Type of aortic valve classification			.30
TAV	47 (90.4%)	78 (95.1%)	
BAV	5 (9.6%)	4 (4.9%)	
Risk scores			
STS‐PROM	9.17 ± 4.5	10.23 ± 5.81	.24
EuroSCORE II (%)	10.62 ± 5.28	12.16 ± 7.52	.17
NYHA functional class			.48
II	1 (1.9%)	2 (2.4%)	
III	22 (42.3%)	24 (29.3%)	
IV	29 (55.8)	56 (68.3%)	

*Note*: Data are presented as mean ± SD or number (%).

Abbreviations: AR, aortic regurgitation; BAV, bicuspid aortic valve; CABG, coronary artery bypass grafting; LVEDD, left ventricular end diastolic dimension; LVEF, left ventricular ejection fraction; LVESD, left ventricular end systolic diameter; NYHA, New York Heart Association; PCI, percutaneous transluminal coronary intervention; TAV, tricuspid aortic valve.

By visually inspecting the CUSUM plots, a decreasing point for total procedure time began at the 52nd operation (Figure S1A), and a similar trend was observed for fluoroscopy time at the 43rd operation (Figure S1B). The mean total procedure time, fluoroscopy time, contrast dye, and procedural characteristics are shown in Table 2. The “technical” learning curve (positioning and implantation) was identified by significantly shorter procedure time and fluoroscopy time, and the use of less contrast dye. The mean total surgical time was 94.22 ± 30.07 in group 1 vs 70.43 ± 15.02 minutes in group 2 (*P* < .05), and the mean fluoroscopy time was 11.52 ± 3.81 in group 1 vs 6.47 ± 1.53 minutes in group 2 (*P* < .05). The mean contrast dye was 84.96 ± 17.76 in group 1 vs 59.95 ± 12.83 mL in group 2 (*P* < .05). There was no significant difference in the device success rates between the two groups (50[96.2%] vs 79[96.3%], *P* = 1.0). The component ratio of larger valve size was significantly greater in the late experience (54[65%] vs 32[61.5%], *P* = .01). All but five of the patients underwent successful devices implantation, and one (1.9%) patient in group 1 required conversion to SAVR for suboptimal angiography imaging leading to valve embolism, and died from stroke 1 month after the operation. One patient had moderate PVL and congestive heart failure 1 week after the operation and who was converted to SAVR, and two (3.7%) patients in group 2 required conversion to SAVR due to one valve migrating into the aortic arch, one valve migrating into the left ventricle, and one coronary obstruction. No second valve was implanted in any case.

**Table 2:** Intraoperative variables

	Group 1 (n = 52)	Group 2 (n = 82)	*P*
Valve size			.01
21/23/25	1/4/14	2/10/16	
27/29	28/4	27/27	
Device success	50 (96.2%)	79 (96.3%)	1.0
Valve in valve	0	1 (1.2%)	.42
Coronary obstruction	0	1 (1.2%)	.42
Conversion to sternotomy	1 (1.9%)	3 (3.7%)	.57
valve migration	0	3 (3.7%)	.16
Valve embolization	1 (1.9%)	0	.21
Contrast dye (mL)	94.22 ± 30.07	70.43 ± 15.02	<.01
Fluoroscopy (min)	11.52 ± 3.81	6.47 ± 1.53	<.01
Total OR time (min)	84.96 ± 17.76	59.95 ± 12.83	<.01

*Note*: Data are presented as mean ± SD or number (%).

Postoperative short‐term outcomes shown in Table 3. The 1‐month survival free from all‐cause mortality and composite endpoint of death and reintervention between the groups were 98.1% and 96.3%, *P* = 1.0, respectively. Three patients died in the group 2; one patient died because of a recurrent severe pulmonary infection and acute kidney injury, another patient died duo to severe digestive tract hemorrhage, and the third patient experienced malignant ventricular arrhythmias resulting in hemodynamic instability. No bleeding required reexploration. No myocardial infarction or vascular complication occurred during the follow‐up after the procedures. According to the Acute Kidney Injury (AKI) Network classification,^11^ one patient in group 2 had stage 3 AKI, and hemodialysis was needed. The number of permanent pacemakers implanted was 5 (9.8%) in group 1 and 7 (8.6%) in group 2 (*P* = 0.83). The extubation in OR rate in group 2 was significantly higher than in group 1 (48[60.8%] vs 0, *P* < .05). The times of mean ICU stay and hospital stay were not significantly different, and an additional extubation ratio rate of ≤24 hours was not different between the two groups.

**Table 3:** Postoperative outcomes

	Group 1 (n = 52)	Group 2 (n = 82)	*P*
Device failure to surgical	1 (1.9%)	0	.21
New postoperative pacemaker	5 (9.6%)	7(8.5%)	.83
Postoperative stroke	0	0	NA
Bleeding requiring rethoracotomy	0	0	NA
Acute kidney injury			.67
Stage 1	6 (11.5%)	12 (14.6%)	
Stage 2	3 (5.8%)	4 (4.9%)	
Stage 3	0	1 (1.2%)	
Extubation ≤24 h	48 (92.3%)	76 (92.7%)	.94
Reintubation	0	4 (4.95%)	.11
Extubation in OR	0	48 (58.5%)	<.01
Postoperative Hospital stay, d	7 (6.8)	6 (5.8)	.21
ICU length of stay, d	1 (1.1)	1 (1.2)	.34
In‐hospital mortality	0	3 (3.7%)	.16
30‐Day mortality	0	3 (3.7%)	.16
Postoperative echocardiographic results			
AV PGmean (mm Hg)	8.24 ± 3.32	8.26 ± 3.35	.98
Peak aortic valve velocity, m/s	1.83 ± 0.29	1.93 ± 0.37	.09
LVEDD, mm	53.68 ± 7.79	57.51 ± 9.08	.01
LVESD, mm	38.08 ± 6.85	43.70 ± 11.03	.02
LVEF, %	53.62 ± 9.88	46.63 ± 12.33	<.01
PVL			.14
None/trivial	33/7 (76.9%)	38/20 (70.7%)	
Mild	11 (21.2%)	22 (26.8%)	
Moderate	1 (1.9%)	0	
Severe	0	0	
NYHA functional class			.01
I	46 (88.5%)	55 (67.1%)	
II	5 (9.6%)	23 (28.0%)	
III	1 (1.9%)	1 (1.2%)	
IV	0	2 (2.4%)	

*Note*: Data are presented as mean ± SD or number (%) or Median (Q1,Q3).

Abbreviations: AV, aortic valve; ICU, intensive care unit; LVEDD, left ventricular end diastolic dimension; LVEF, left ventricular ejection fraction; LVESD, left ventricular end systolic diameter; NYHA, New York Heart Association; PVL, paravalvular leakage.

## 5 ECHOCARDIOGRAPHIC FINDINGS AND NYHA FUNCTIONAL CLASS

The results of the echocardiogram follow‐up are shown in Table 3. Transthoracic echocardiograms were performed 1 month after surgery. Moderate PVL was found in one patient in group 1; the frequency of mild PVL was not significantly decreased in the second group (11 [21.6%] vs 22 [27.8%]; Figure S3). The average LVEF was significantly better in group 1 (53.62 ± 9.88 vs 46.63 ± 12.33%, *P* < .01). Mean aortic valve gradient (8.24 ± 3.38 vs 8.26 ± 3.35 mm Hg, *P* = .98). The enlarged left ventricle, indicated by both LVEDD (64.62 ± 8.55 vs 65.52 ± 9.02 mm, *P* = .56) to (53.68 ± 7.79 vs 57.51 ± 9.08 mm, *P* = .01) and LVESD (44.81 ± 9.52 vs 47.89 ± 11.40 mm, *P* = .56) to (38.08 ± 6.85 vs 43.70 ± 11.03 mm Hg, *P* = .02) were reduced significantly. Nearly 98% of patients experienced NYHA functional class III or IV symptoms before the procedure, and most of the patients with NYHA functional significantly improved to class I or II, 1 month after surgery (Figure S2).

## 6 DISCUSSION

Patients with severe AR have a higher risk of symptomatic deterioration, increasing the probability of symptoms at a rate of 25% per year and death at an annual rate of 10% to 20%.^12^ SAVR can have considerable influence on cardiopulmonary function, especially for elderly patients with many comorbidities.^13^ TAVR is being increasingly performed for native AR. This is likely due to a combination of operator experience and technological advances. Procedural complications seem to reduced, although some critical complications remain. TAVR can reduce surgical trauma, but performing it in AR patients is technically demanding. Our previous outcome using the J‐valve prosthesis for AR is excellent.^14^ However, when a new technique is developed, a learning curve is needed for a surgeon to gain experience and confidence.

Transcatheter aortic valve replacement has been used for native AR patients in recent years, and studies have demonstrated that TAVR using new‐generation devices was associated with a significantly high device success rate and low adverse event rate in treating patients with native AR.^15^ An international registry study of TAVR in the treatment of Pure AR in native valves vs failing surgical bioprostheses indicated that TAVR is a valuable therapeutic option for AR.^16^ No report has been published regarding the number of operations that a surgeon is required to have participated in to become proficient with AR.

Surgeons became more proficient in TAVR procedures, and measures of the learning curve effect improved as the number of procedures performed increased. There was an apparent decrease in intraprocedural times and significant decreases in contrast and radiation dose with increasing case volumes. This study also shows that this was true for TAVR with AS; a definite procedure‐related learning curve was evidenced by our decreased procedural and fluoroscopy times with reduced contrast volume and complications.^17^, ^18^ A systematic review and meta‐analysis indicated that TAVR appears to be a viable option for high surgical risk or inoperable patients with native AR.^19^ The lack of standardized techniques, the relatively limited lack of aortic valve calcification, and the subsequent difficulty in anchoring make the learning experience of surgeons interested in TAVR surgery problematic. Surgeons might experience more postoperative morbidity events or a higher conversion rate associated with a longer procedure time, so some number of cases must be deemed to represent a training period before an objective evaluation of the benefits of a new procedure is undertaken. A small case study failed to demonstrate the superiority of a promising TAVR technique. According to Sergey Gurevich et al establishing a partnership with an established program can help mitigate the learning curve associated with these complicated procedures.^20^ Therefore, analyzing the learning curve for TAVR for AR not only benefits novice surgeon training but also helps in the accurate evaluation of the advantages of TAVR.

Transcatheter aortic valve replacement prostheses can be implanted using the antegrade transapical (TA‐TAVR) approach or retrograde trans‐vascular access (TF‐TAVR). TA‐TAVR access was more frequently used for the second‐generation device in AR patients.^15^, ^19^ According to surgical proficiency, TF‐TAVR can be achieved with approximately 30 cases for surgeons experienced in AS with TAVR.^4^, ^14^ Takahide Arai et al reported that the CUSUM analysis revealed a learning curve with improvement after the initial 86 cases using the Edwards valve and 40 cases using the CoreValve regarding the occurrence of 30‐day adverse events,^21^ which may be the type of valve influencing the learning curve. In another study by Oluseun et al involving 44 cases of AS with TAVR, the operative time was significantly reduced after 30 cases, and the radiation and contrast volumes decreased significantly showing increasing proficiency with evidence of plateau after the first 30 cases.^22^ TA‐TAVR has more technical requirements; for example, in the PARTNER‐I trial, the learning curve to achieve technical efficiency for TA‐TAVR requires 30 to 45 procedures.^5^ AR is frequently associated with large annular anatomy and a dilated ascending aorta, and the absence of aortic annular calcification means more technical requirements. Since creation of TAVR for AR is more complex than TAVR in the AS, we had to analyze the learning curve of the technical procedure.

When analyzing the learning curve the CUSUM proved particularly valuable in helping to intuitively discern the trend of a data set.^10^ This method has been widely used to calculate the required number of cases to gain experience with a new technique.^23^ In our study, the CUSUM plots of procedure time and fluoroscopy time present their inflection points at the 52nd case and the 43rd case, respectively, and imply that approximately 52 cases are needed to gain early proficiency in TAVR for AR for surgeons who are familiar with standard cardiovascular surgical procedures. The time required for chest incision, fluoroscopy, location, and instrument exchange is cumbersome in TAVR surgery. Therefore, the proficiency of the team is critical to the entire surgical procedure. However, to the best of our knowledge, there have been no reports on the TAVR learning curve with AR until now. By visually inspecting the CUSUM plots of our cases, contrast dye usage also decreased significantly after performing 52 operations.

Since the TAVR procedure was technically demanding and time‐consuming, some surgeons preferred TF‐TAVR for aortic valve disease during the early stage of the learning curve.^4^ We found a decreasing point for TAVR in AR console time in case 52, which seemed more difficult for AS patients. Some of the reasons that may result in selection bias included challenging cases for surgeons beginning a new surgical procedure who have no prior experience with TAVR, difficulty identifying the annular plane, a lack of calcification that may lead to PVL, the TAVR devices that were designed to treat aortic stenosis, and a more complex and variable anatomy insufficient with radial support. By visually inspecting the CUSUM plots of our results, a decreasing point for fluoroscopy time presented at case 43, even earlier than that for the procedure time. Therefore, in our opinion, the apical incision might be a promising alternative technique to procedure time or fluoroscopy time when the surgeon has completed the learning phase.

Although a significant decrease in the procedure time, fluoroscopy time, and contrast dye were noted after the first 52 cases, the device success, 30‐day mortality, postoperative hospital length of stay and the frequency of major comorbidities were not significantly different between the two groups, which revealed that using the J‐valve prosthesis for TAVR with AR was a relatively safe and feasible procedure even during the early‐experience stage. The morbidity and perioperative outcomes in the two groups were acceptable, and whether surgeons performing TAVR on patients with AR have a learning curve similar to that reported for the same procedure on patients with AS is not known. Even though the NYHA functional class seems worse after surgery in group 2, accordingly, TAVR for AR might be an alternative, revealing that group 2 had a higher STS score, which was lower in LVEF, larger in annular size, and might make it more challenging for the TAVR procedure. However, there was no significant difference in all‐cause mortality, the incidence of adverse events, the time to rehabilitation, valve function, NYHA functional classification in these two groups. This means that after achieving the TAVR, the technical efficiency may reduce the incidence of adverse events. Even though extubation in the OR was significantly higher in group 2, the early outcome of cardiac rehabilitation may be related to the surgeon's experience. All operations in this study were performed by a single team experienced in both SAVR and TAVR. The benefit of such a design reduced the deviation resulting from the heterogeneity of the surgical approach, postoperative care, or data collection.

## 7 LIMITATIONS

There are some limitations including the retrospective nature of the analysis, operation and procedure classification system, which allows TA‐TAVR procedures to be performed by a single team. The sample size was relatively small and long‐term outcomes were not available. In addition, we focused on surgical time for analysis since operation time is the most widely used marker for the learning curve in our report. Conversions, postoperative morbidity, mortality, apical incision, and cardiac function rehabilitation were also important indicators of a learning curve. In the procedure time parameter for these AR cases, we including cases performed for aortic stenosis that may affect our learning curve. Therefore, extrapolating that the learning curve is approximately 50 cases maybe not precise because we practiced by doing other similar procedures, but our experience may reflect a real‐world treatment learning curve.

## 8 CONCLUSIONS

For a surgeon without previous TAVR experience, 52 cases of performance is the minimal requirement to gain the proficiency of TAVR for native AR. The skilled surgeons have been observed with reduced procedural time, fluoroscopy times, radiation exposure dose, and contrast volume usage. However, the overall prognosis was not significantly different between the two groups.

## CONFLICT OF INTEREST

The authors declare no potential conflict of interests.

## Supporting information


**Figure S1** By visually inspecting the CUSUM plots, A) a decreasing point for procedure time begins at the 52th operation; B) a decreasing point for fluoroscopy time begins at the 43th operation; C) The trend chart of operative time (OT) of TAVR for AR; D) The trend chart of fluoroscopy time (FT) of TAVR for AR

**Figure S2** Symptom status for two groups with matched data sets at all time points are presented.
NYHA = New York Heart Association.

**Figure S3** The number of patients by degree of PVL at 30 days follow‐up.
PVL = Paravalvular leakage.
