# Supplementary figures and images for: Learning curve for transcatheter aortic valve replacement for native aortic regurgitation: Safety and technical performance study
Source: Clin Cardiol. 2020 Jan 11;43(5):475–82. doi: 10.1002/clc.23332 (PMC7244294; doi:10.1002/clc.23332)

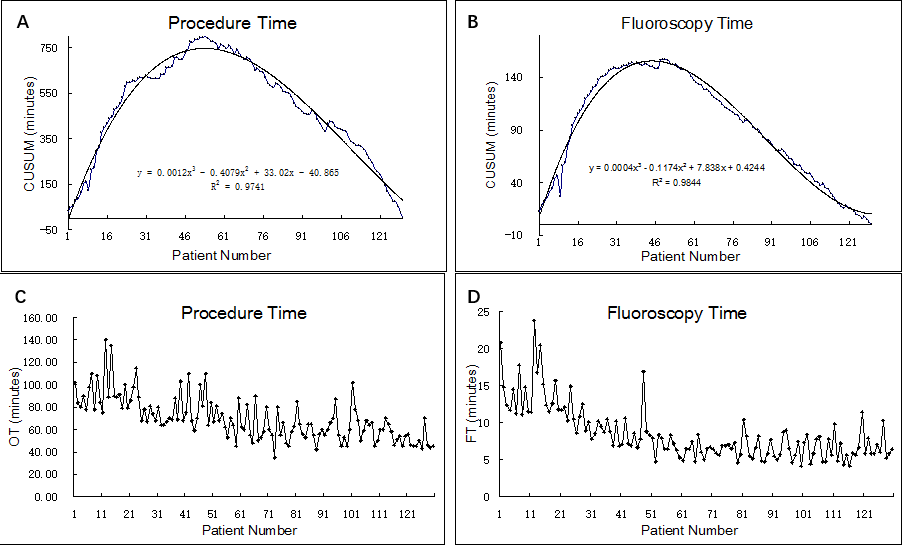

Supplement: Supplementary file 1 — Figure S1 By visually inspecting the CUSUM plots, A) a decreasing point for procedure time begins at the 52th operation; B) a decreasing point for fluoroscopy time begins at the 43th operation; C) The trend chart of operative time (OT) of TAVR for AR; D) The trend chart of fluoroscopy time (FT) of TAVR for AR [file CLC-43-475-s001.tif]

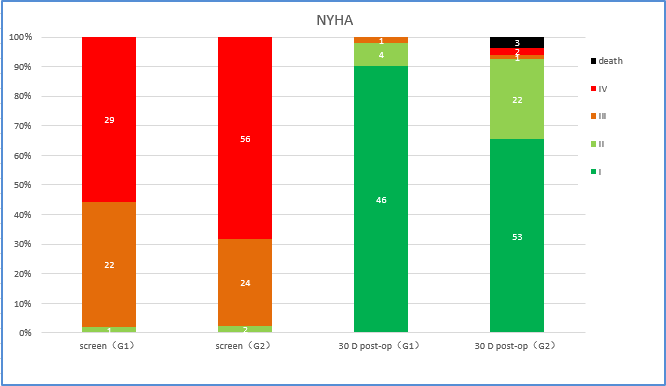

Supplement: Supplementary file 2 — Figure S2 Symptom status for two groups with matched data sets at all time points are presented. NYHA = New York Heart Association. [file CLC-43-475-s002.tif]

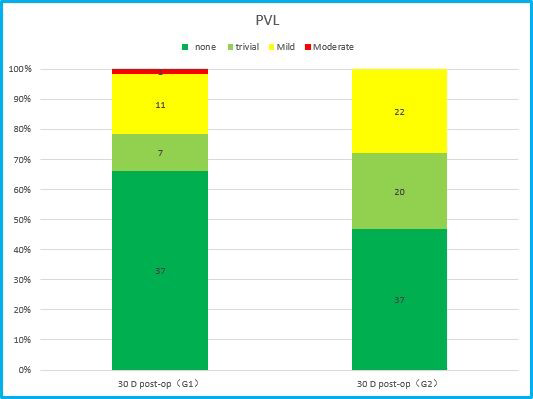

Supplement: Supplementary file 3 — Figure S3 The number of patients by degree of PVL at 30 days follow‐up. PVL = Paravalvular leakage. [file CLC-43-475-s003.tif]
